# ASO Author Reflections: The Role of Physiotherapy Regimens in Esophagectomy and Gastrectomy for Cancer

**DOI:** 10.1245/s10434-021-11163-y

**Published:** 2021-12-11

**Authors:** Karina Tukanova, Swathikan Chidambaram, Nadia Guidozzi, George B. Hanna, Alison H. McGregor, Sheraz R. Markar

**Affiliations:** 1 Division of Surgery, Department of Surgery and Cancer, Imperial College London, London, UK; 2 Department of Surgery, University of the Witwatersrand, Johannesburg, South Africa; 3 Nuffield Department of Surgery, University of Oxford, Oxford, UK; 4 Department of Molecular Medicine and Surgery, Karolinska Institutet, Solna, Sweden

## PAST

Despite advancements in surgical management, esophageal and gastric cancer surgery is still associated with a significant morbidity. Traditionally, esophagectomy and gastrectomy via an open surgical approach has been the treatment of choice for esophageal and gastric cancer, respectively.^1,2^ Enhanced recovery after surgery (ERAS) protocols commonly include physiotherapy regimens or early mobilization intervention. These programs are well-established in colorectal cancer surgery and have shown to reduce postoperative complication rates and shortened the length of hospital stay (LOS).^3^

Only a small number of studies have assessed the role of respiratory physiotherapy in gastrointestinal cancer surgery, while this patient group commonly present with pre-existing respiratory disease and is particularly at risk for malnutrition and loss of muscle mass.^4^ Although there is growing evidence of the benefits of physiotherapy implementation in decreasing the risk for postoperative morbidity, there is currently insufficient strong evidence for routine implementation of standardized respiratory physiotherapy in esophageal and gastric cancer surgery.

## PRESENT

This is the first meta-analysis assessing the effect of prehabilitation and peri- or postoperative physiotherapy regimens on postoperative mortality and morbidity in esophageal and gastric cancer surgery.^5^ A lower incidence of pneumonia was observed following both prehabilitation and peri- or postoperative rehabilitation. Furthermore, a lower incidence of postoperative morbidity was seen in patients undergoing prehabilitation, while peri- or postoperative rehabilitation resulted in a shorter LOS and better health-related quality-of-life scores for dyspnea and physical functioning. These results suggest that implementation of a physiotherapy regimen in both the pre- and peri- or postoperative setting may be beneficial. This meta-analysis is however limited by the lack of a standardized physiotherapy protocol for patients undergoing esophagectomy or gastrectomy, and by the lack of functional data in long-term survivors.

## FUTURE

Further research is required focusing on the mechanism in which surgery might contribute to the development of postoperative complications and what outcomes are most likely to be affected. This information may aid in identifying which components of physiotherapy regimens have the greatest impact on clinical outcomes. In the future, tailored physiotherapy regimens may be developed aimed at decreasing the risk for complications in the preoperative setting (prehabilitation) and improve clinical outcomes postoperatively (rehabilitation) in major gastrointestinal cancer surgery.

## Discloure

Karina Tukanova, Swathikan Chidambaram, Nadia Guidozzi, George B. Hanna, Alison H. McGregor, and Sheraz R. Markar declared no conflicts of interest.
